# Demonstration of Ultraviolet Photodissociation Induced by Light-Emitting Diodes Externally Interfaced to a Linear Ion-Trap Mass Spectrometer

**DOI:** 10.1021/jasms.6c00354

**Published:** 2026-10-05

**Authors:** Bricia M. Parreiras, Sarah A. Wilson, Pongpanot Pongworasuwanna, Ruby Spratt, Jackie A. Mosely, Caroline E. H. Dessent

**Affiliations:** Department of Chemistry, 8748 University of York, YO10 5DD York, United Kingdom

## Abstract

We report the first demonstration of ultraviolet photodissociation (UVPD) mass spectrometry achieved with light-emitting diodes (LEDs) externally interfaced to a linear ion-trap mass spectrometer. LEDs provide a low-cost, compact photon source that is technologically simpler to operate than the high-powered pulsed laser systems that are currently used in UVPD instruments. Mounting the LEDs external to the mass spectrometer provides improved flexibility for interchanging LEDs with different wavelengths. Here, we demonstrate 365 and 280 nm LED-UVPD on flavin mononucleotide, tryptophan, and the pentapeptide Leu-Enk. The UVPD fragments observed are in full agreement with fragments observed using pulsed lasers as the photon source.

## Graphical abstract

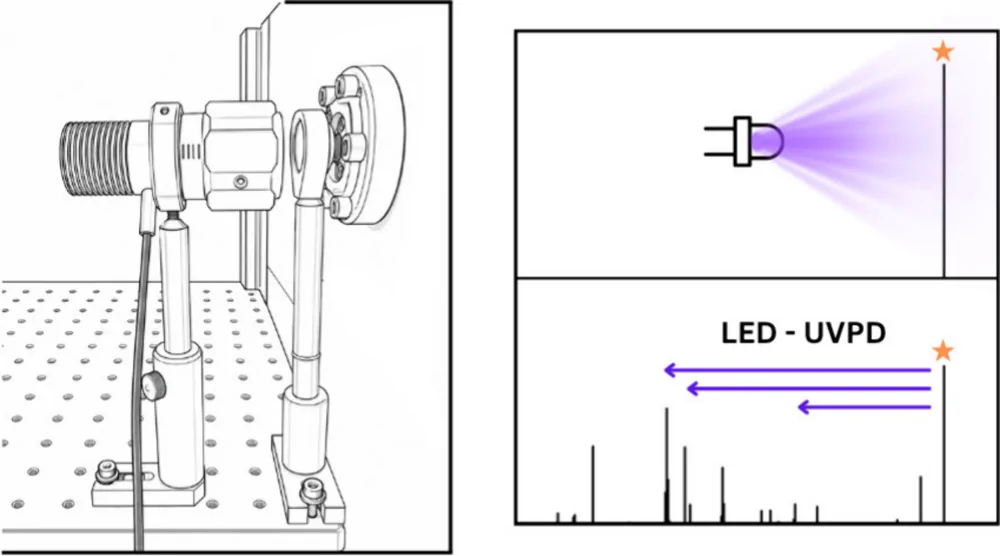

## Introduction

Tandem mass spectrometry techniques for structure elucidation rely on initiating molecular fragmentation via ion activation. A key ongoing goal in mass spectrometry is therefore the development of new ion activation techniques that can facilitate enhanced structural characterization.^1^ While traditional, lower-energy collisional methods (e.g., collision-induced dissociation) are still widely employed, there have been significant recent advances using higher-energy techniques, including ultraviolet photodissociation (UVPD). UVPD has been demonstrated to be particularly valuable for the analysis of proteins and peptides, where photons excite chromophores within aromatic amino acids of the peptide backbone.^2^ Fragmentation is rapid, occurs on sub-microsecond time scales, and accesses high-energy fragmentation routes involving the formation of a-, c-, x-, and z-fragment ions.^3^ It has proven exceptionally useful for identifying post-translational modifications (PTMs), since the rapid fragmentation time scales maintain the integrity of fragile PTMs.^4^ UVPD has also been demonstrated to be of high utility for analysis of small molecules including pharmacophores, organic micropollutants, and agrichemicals where isomeric structures can be difficult to distinguish via low-energy collisional dissociation.^5–7^

While the considerable potential of UVPD as an advanced fragmentation technique has been convincingly demonstrated, its wide-scale take up is hampered by the high cost of the current photon sources employed in commercially available UVPD mass spectrometry (MS) instruments. These are generally nanosecond-pulsed lasers, operating at 213 nm. In addition to being expensive, lasers require personnel with significant technical expertise to perform maintenance and vary photon wavelengths, which introduce operational inflexibility. In an attempt to overcome some of these issues, Holden et al. demonstrated the potential of light-emitting diodes (LEDs) as an alternative photon source for UVPD.^8^ LEDs offer many advantages, primarily in terms of cost, size, and robustness. In the instrumental approach used by Holden et al., multiple LEDs were mounted within the trapping cell of an Orbitrap mass spectrometer. This solution was adopted due to the low energy of the UV-LEDs available at the time, requiring multiple LEDs in close proximity to the ions to achieve photodissociation. Despite having demonstrated proof-of-principle, the approach has not subsequently moved forward to commercialization or wide-scale uptake by the MS community.

Over the time since Holden *et al.* used LEDs for UVPD in an Orbitrap MS, LED technology has improved, with output power at wavelengths below 300 nm increasing significantly.^9^

Here, we take advantage of the improved output power of LEDs to demonstrate LED-UVPD on a commercial MS platform with the photon sources located external to the vacuum of the MS instrument. This technical approach is straightforward to set up, allows for easy switching between different LED photon energies, and demonstrates a clear pathway for the development of fully commercial LED-UVPD platforms.

## Experimental Section

All compounds were prepared in methanol/water (50:50, v/v) to final analyte concentrations of 20 μM. Samples were introduced to the mass spectrometer by direct infusion using electrospray ionisation, and studied in positive ion mode. Experiments were conducted in a modified commercial mass spectrometer, LTQ XL (Thermo Fisher Scientific, Inc, USA), which had been adapted to provide optical access to the ion-trap region for photodissociation.^10,11^ UV photons were provided by two LED light sources; a 365 nm ultraviolet LED (M365L3, Thorlabs, Newton, NJ, USA) and a 280 nm ultraviolet LED (M280L6, Thorlabs, Newton, NJ, USA). The output power of the 365 nm diode used here is a factor of 10 greater than the 280 nm diode (1.29 W versus 114 mW maximum power, respectively). Low-energy collision induced dissociation (CID) experiments were conducted using standard instrumental conditions. Further experimental details, including optical setup (Figure S1), beam dimensions, power measurements (Table S1), and irradiation-time measurements (Figures S2 and S3), are provided in the Supporting Information.

## Results and Discussion

Initial evaluation of the ability of the externally mounted LEDs to effect UVPD was conducted on flavin mononucleotide (FMN). This molecule is highly conjugated and absorbs strongly across the visible and UV spectral regions, with a maximum aqueous-phase absorption wavelength of 268 nm. It was used previously as a prototype molecule to demonstrate UVPD by Bellina *et al.* ^12^ and Holden *et al*.^8^

Figure 1A displays the MS/MS CID spectrum of [FMN·H]^+^ obtained following isolation of the base peak ion (*m/z* 457). We observe primary CID fragments at *m/z* 439 (loss of water), *m/z* 414 (loss of HNCO), *m/z* 359 (loss of a phosphate group) and *m/z* 243 (loss of the ribose side chain) which agree well with previous reports.,^8,12^

**Figure 1:**
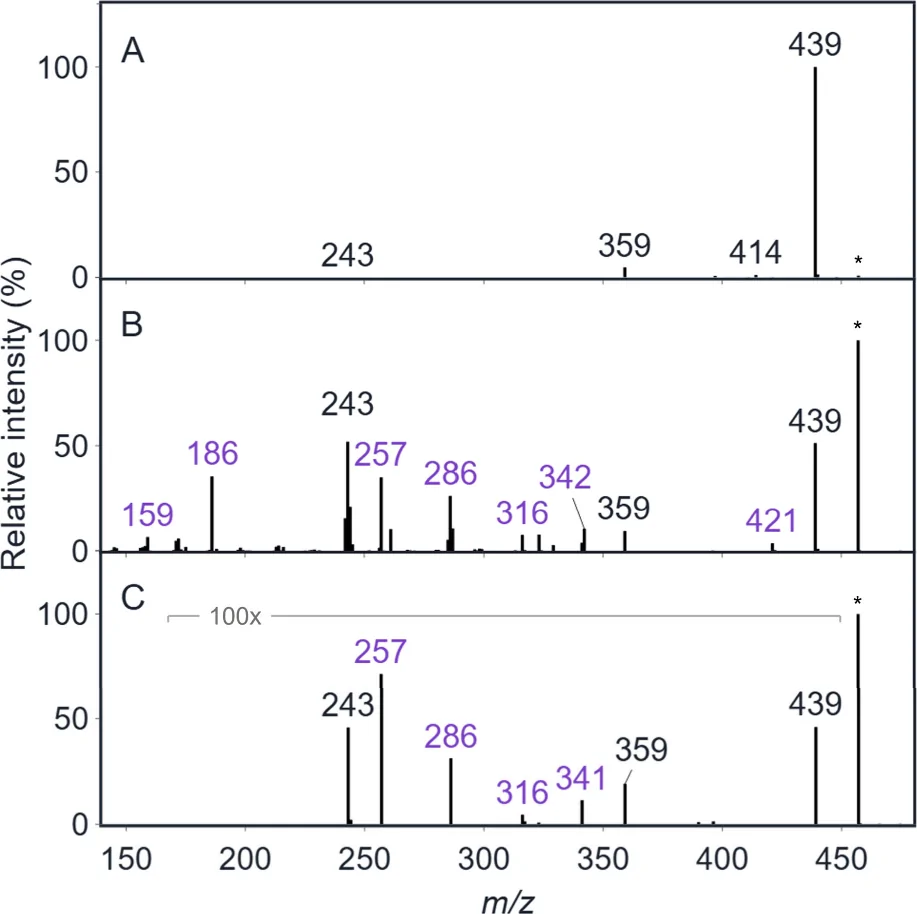
A) MS/MS spectrum of [FMN·H]^+^ with 20% CID energy (30 ms activation time), B) LED-UVPD of [FMN·H]^+^ using the 365 nm LED (10 s irradiation), and C) LED-UVPD of [FMN·H]^+^ using the 280 nm LED (10 s irradiation). * indicates the precursor ion (*m/z* 457). Purple *m/z* labels indicate photofragment ions detected with UVPD, but not with CID.

Figures 1B and 1C display the MS obtained when [FMN·H]^+^ is isolated, and the UV-LEDs (365 and 280 nm, respectively) are introduced to the ion-trapping region to irradiate the precursor ion prior to mass analysis. UVPD is observed with both wavelength LEDs. For 365 nm UVPD, it is possible to achieve greater than 70% photodepletion of the precursor ion and extensive photofragmentation is observed, with major ions including *m/z* 439, 359, 342, 286, 257, 243, and 186. Two points are of note: First, while there are common UVPD and CID fragments (*m/z* 439, 359, and 243), their relative intensities differ in UVPD versus CID. Secondly, new fragment ions which are not present with CID are observed, for example *m/z* 257 (lumiflavin) and *m/z* 286 (formylmethylflavin). These ions are typical photoproducts of flavin compounds.,^13,14^ With the 280 nm diode, approximately 2% photodepletion of the precursor ion is achieved, with production of *m/z* 439, 359, 341, 286, 257, and 243 as the major fragments. This set of photofragments (280 nm) matches well with those observed for UVPD by Bellina *et al*. (using a 266 nm Continuum Minilite 10 Hz pulsed laser) and by Holden *et al*. (using a bank of 8 LEDs located within the trapping cell of a Thermo Elite Orbitrap mass spectrometer). The results shown in Figure 1 therefore demonstrate the proof-of-principle that external UV-LED light sources can achieve UVPD equivalent to using a pulsed laser source or diodes mounted within a highly customised orbitrap instrument. Table S2 lists the fragment ions and assignments for [FMN·H]^+^, with Table S3 comparing our results to Holden *et al*.^8^

LED-photodissociation involves relatively low photon fluxes compared to traditional pulsed laser systems, so that it is necessary to subject trapped ions to sufficient irradiation times to achieve acceptable levels of photofragmentation (Figures S2 and S3). Using the higher-power 365 nm diode, 20% photodepletion of [FMN·H]^+^ could be achieved with a 1 s irradiation time, whereas reasonable levels of photofragment ion production required 10 s of irradiation time for the lower-power 280 nm diode. For both diodes, the majority of photofragments displayed intensities which increase linearly as a function of irradiation time. For the 365 nm diode, there is evidence of secondary photofragmentation at long irradiation times (> 2 s); this provides an explanation of the presence of the lighter mass *m/z* 159 and 186 ions in the 365 nm UVPD compared to the 280 nm spectrum. We note that UVPD fragments observed with 365 and 280 nm photoexcitation will also differ due to different bright electronic transitions being accessed at the two photoexcitation energies with associated distinctive photodissociation pathways.^15^

We performed a number of further measurements to explore the generality of achieving UVPD with LEDs mounted external to the MS instruments. Figure 2 displays 280 nm LED-UVPD results obtained for protonated tryptophan [TRP·H]^+^, and the protonated pentapeptide, [Leu-Enk·H]^+^. LED-UVPD of [TRP·H]^+^ (Figure 2B) results in the production of a set of fragments (*m/z* 188, 170, 159, 146, 144, 132, 130) that differ significantly from CID MS/MS fragmentation which produces predominantly *m/z* 188 with loss of NH_3_ (Figure 2A). The LED-UVPD fragments we observe are again in excellent agreement with those observed in previous pulsed-laser 284 nm photodissociation experiments of [TRP·H]^+^.^16,17^ (Interestingly, *m/z* 188 was observed to dissociate into *m/z* 146 at longer detection times following pulsed laser excitation.^17^) Table S4 lists the fragments and assignments for [TRP·H]^+^.

**Figure 2:**
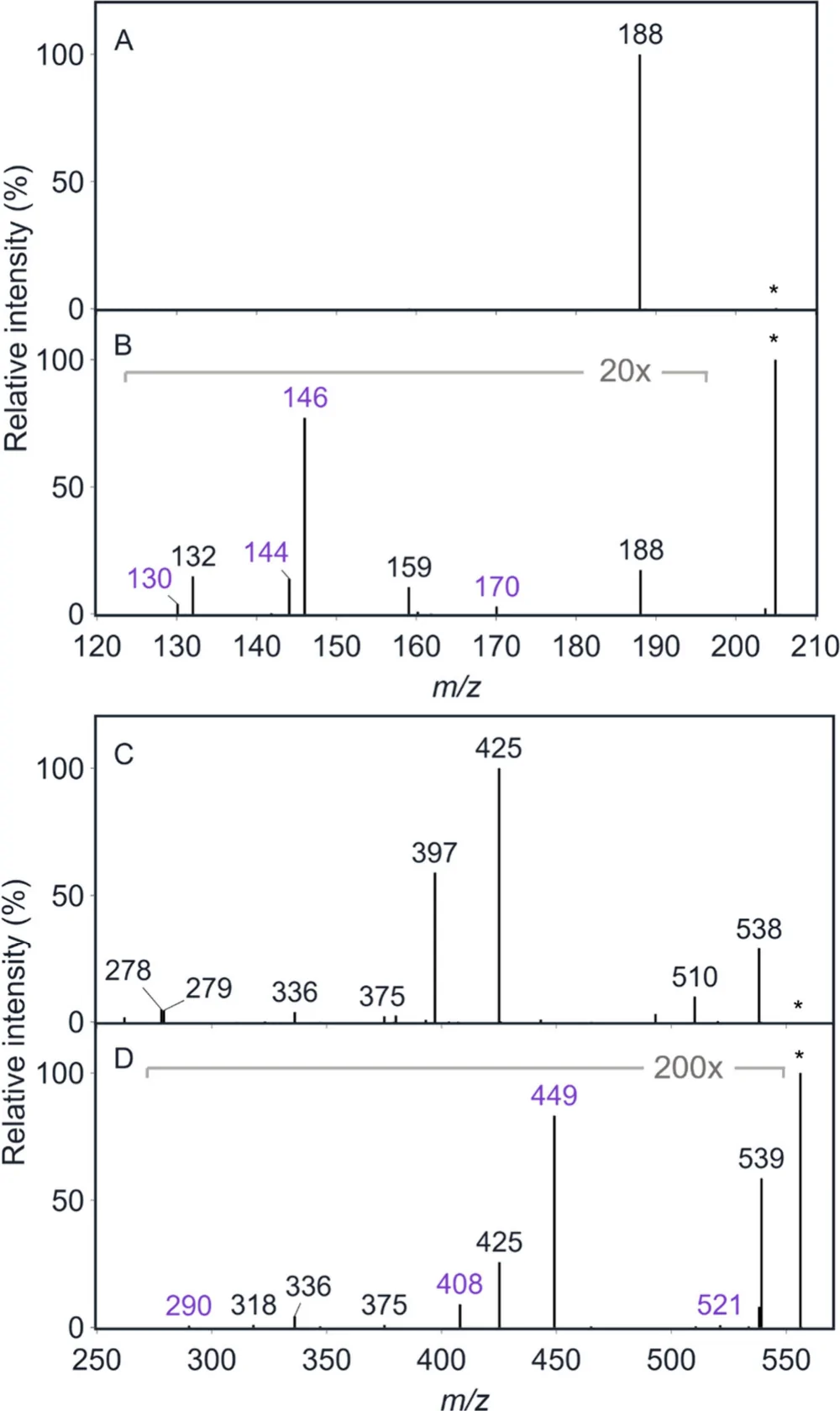
A) MS/MS spectrum of [TRP·H]^+^ (*m/z* 205) with 20% CID energy (30 ms activation time), B) LED-UVPD of [TRP·H]^+^ using the 280 nm LED (irradiation time of 10 s), C) MS/MS spectrum of [Leu-Enk·H]^+^ with 23% CID energy (30 ms), and D) LED-UVPD of [Leu-Enk·H]^+^ using the 280 nm LED (irradiation time of 10 s). * indicates the precursor ions. Purple *m/z* labels indicate product ions detected in the UVPD spectra but not with CID.

Finally, we performed UVPD on the pentapeptide leucine enkephalin, Leu-Enk (Tyr-Gly-Gly-Phe-Leu). CID of protonated Leu-Enk, [Leu-Enk·H^+^], produced *m/z* 538, 510, 425, and 397 as the major MS/MS fragments (Figure 2C). In contrast, 280 nm LED-UVPD produced *m/z* 539, 449, and 425 as the most intense UVPD fragments. These fragments were observed as the most intense photofragments in previous 280 nm pulsed-laser UVPD of [Leu-Enk·H^+^], and correspond to ammonia loss (*m/z* 539), loss of a 107 amu unit (*m/z* 449) and b_4_ fragmentation.^18,19^ The *m/z* 449 fragment is notable as it is a purely photochemical fragment that results from loss of the neutral tyrosine side chain, given that 280 nm excitation is expected to predominantly excite the tyrosine (Tyr) chromophore.^2,3^ The lower intensity UVPD fragments (e.g. 408, 375, 336) were also all observed in previous pulsed-laser UVPD of [Leu-Enk·H]^+^.^18,19^ The excellent agreement between the LED-UVPD results displayed in Figure 2D and the previous pulsed-laser photodissociation measurements demonstrates the proof-of-principle that LED-UVPD will be applicable to peptides. Table S5 gives a list of the LED-UVPD fragments observed with assignments.

## Conclusions

The results presented above demonstrate for the first time that LEDs mounted external to the instrument body of a mass spectrometer can be used to perform UVPD of small biological molecules and a pentapeptide. This represents a significant advance, since minimal instrument modification of a commercial mass spectrometer is needed to achieve external LED-UVPD, and LED photon sources are substantially cheaper than currently employed pulsed-laser systems. While improvements in LED output power for UV wavelengths below 280 nm are still needed to achieve the fullest UVPD coverage (e.g., to perform widely used 213 nm UVPD^2^), our results demonstrate proof-of-principle that externally mounted LEDs can replace pulsed laser systems, thus enabling much wider uptake of UVPD technology than is currently possible. This is an important advance for structural biology MS given the utility of UVPD in characterizing post-translational modifications,^4^ as well as for isomer differentiation in small molecules analysis.^5–7^

## Supplementary Material



## Data Availability

Data for the results reported are available at 10.15124/07d3137a-4d28-41fb-a40f-479ba5474ac3.

## Notes

Materials and Methods (Section S1); optical power measurements outside the instrument and after transmission through the ion-trap region (Table S1); optical setup and beam characterisation (Figure S1); irradiation-time-resolved fragment-ion plots for FMN using the 365 and 280 nm LEDs (Figures S2 and S3); fragment ions observed for CID (MS/MS) and LED-UVPD (365 and 280 nm) of FMN (Table S2); comparison of fragment ions reported by Brodbelt and co-workers with those observed in this work (Table S3); comparison of fragment ions reported by Molina et al. with those observed in this work using CID and LED-UVPD (280 nm) of protonated tryptophan (Table S4); comparison of fragment ions reported by Herburger et al. with those observed in this work using CID and LED-UVPD (280 nm) of protonated Leu-Enk (Table S5) (PDF)
