# Supplementary material for: Demonstration of Ultraviolet Photodissociation Induced by Light-Emitting Diodes Externally Interfaced to a Linear Ion-Trap Mass Spectrometer
Source: J Am Soc Mass Spectrom. 2026 Oct 5;37(11):2674–7. doi: 10.1021/jasms.6c00354 (PMC13741918; doi:10.1021/jasms.6c00354)
Supplement: Supplementary file 1 [file js6c00354_si_001.pdf]

Supporting Information

Demonstration of Ultraviolet Photodissociation Induced by Light-Emitting Diodes Externally Interfaced to a Linear Ion-Trap Mass Spectrometer

Bricia Parreiras, Sarah A. Wilson, Pongpanot Pongworasuwanna, Ruby Spratt, Jackie A. Mosely,* and Caroline E. H. Dessent*

Department of Chemistry, University of York, YO10 5DD, York, United Kingdom.

* Corresponding authors. E-mail: caroline.dessent@york.ac.uk; jackie.mosely@york.ac.uk

- Section S1. Materials and Methods
- Figure S1: Optical setup used to couple the LED beam into the mass spectrometer, showing the beam transmitted through the ion-trap region.
- Figure S2: Fragment-ion intensity versus irradiation time for selected FMN photoproduct ions using the 365 nm LED.
- Figure S3: Fragment-ion intensity versus irradiation time for selected FMN photoproduct ions using the 280 nm LED.
- Table S1: Optical power measured outside the instrument at approximately 13 cm from the final focusing lens and after transmission through the ion-trap region.
- Table S2: Fragment ions observed for CID (MS/MS) and LED-UVPD (365 and 280 nm) of FMN.
- Table S3: Comparison of fragment ions observed for protonated FMN by Brodbelt and co-workers (Ref [3]) and the current work using CID (MS/MS) and LED-UVPD (365 and 280 nm).
- Table S4: Comparison of fragment ions reported by Molina et al. (Ref [4]) with those observed in this work using CID (MS/MS) and LED-UVPD (280 nm) of protonated tryptophan.
- Table S5: Comparison of fragment ions reported by Herburger et al. (Ref [5]) with those observed in this work using CID (MS/MS) and LED-UVPD (280 nm) of protonated Leu-Enk.

Section S1: MATERIALS AND METHODS

Materials

Flavin mononucleotide monosodium salt dihydrate (FMN) was obtained from AppliChem GmbH. L-Tryptophan and leucine enkephalin (Leu-Enk, Tyr-Gly-Gly-Phe-Leu) were obtained from Sigma-Aldrich and used without further purification. Optima LC/MS-grade methanol, water and formic acid were obtained from Fisher Chemical. Solutions were prepared at an analyte concentration of 20 µM in methanol/water (50:50, v/v) containing 0.1% (v/v) formic acid.

Mass spectrometry

Samples were introduced by direct infusion and analysed using positive-mode electrospray ionization on an LTQ XL linear ion trap mass spectrometer (Thermo Fisher Scientific, San Jose, CA, USA). The instrument was modified to provide optical access to the linear ion trap, and the optical alignment was performed as described previously by Dang et al. [1]. FMN, tryptophan and Leu-Enk were analyzed as $[FMN+H]^+$ at *m/z* 457.11, $[Trp+H]^+$ at *m/z* 205.1 and $[Leu\text{-}Enk+H]^+$ at *m/z* 556, respectively.

CID spectra were acquired using an activation time of 30 ms. FMN and tryptophan were fragmented using 20% normalized collision energy, while Leu-Enk was fragmented using 23% normalised collision energy.

LED-UVPD

UV radiation was provided by a 365 nm LED (M365L3, Thorlabs, Newton, NJ, USA) or a 280 nm LED (M280L6, Thorlabs). The LEDs were operated in continuous-wave mode using a Thorlabs LEDD1B constant-current driver. The 365 and 280 nm LEDs were operated at their maximum rated drive currents of 1.0 and 0.5 A, respectively.

Optical access to the linear ion trap follows Ref [1]. To focus the LED beam into the mass spectrometer, we used a Thorlabs SM1U25-A collimation adaptor, modified with a f = 30 mm UV Fused Silica lens to collimate the highly divergent (112°) LED beam, and an MgF2 f = 100 mm lens to focus into the trap. Irradiation times ranging from 10 to 60,000 ms were investigated. LED-on and LED-off spectra were acquired using identical precursor-isolation and ion-storage conditions at each irradiation time. For the LED-off controls, the precursor ions were stored for the selected period without exposure to UV radiation.

Optical Characterization

The optical set-up was designed to focus the LED output into the ion-trap region. To assess the beam transmitted through the trap, the emerging UV beam was visualized on a beam card positioned on the opposite side of the ion-trap region. The transmitted beam had an approximate diameter of 4 mm.

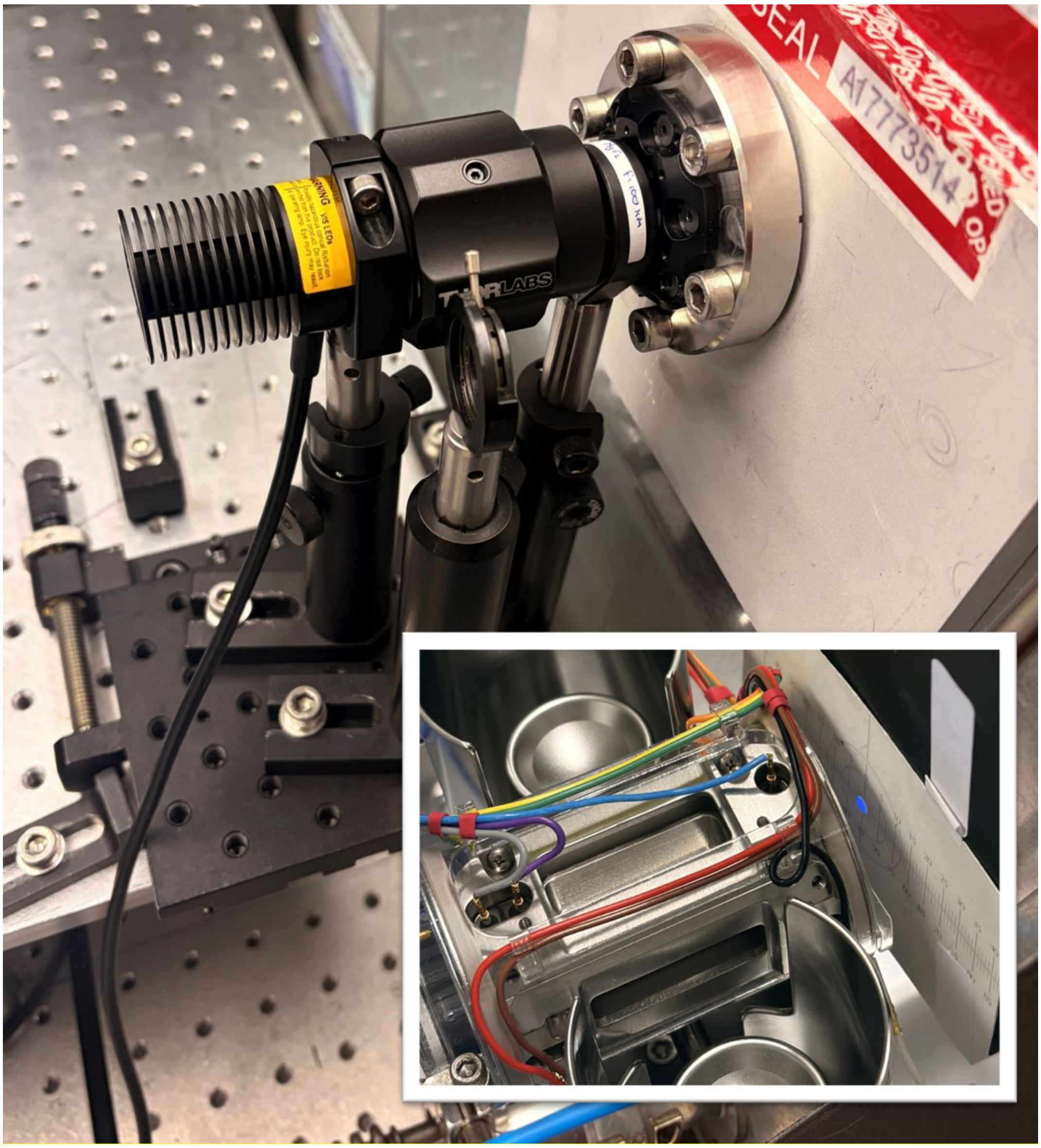

Figure S1. Optical set up used to couple the LED beam into the mass spectrometer. The insert shows the beam transmitted through the ion-trap region and emerging on the opposite side, with an approximate diameter of 4mm.

Power Measurements

Optical power measurements were collected at matched drive currents immediately after the ion-trap region and outside the instrument. The mean background-corrected power measured after the trap region was $(7.62 \pm 0.08) \times 10^{-4}$ W for the 365 nm LED and $(6.86 \pm 0.11) \times 10^{-5}$ W for the 280 nm LED, corresponding to 762 ± 8 μW and 68.6 ± 1.1 μW, respectively. The corresponding outside-instrument powers were $(1.3219 \pm 0.0038) \times 10^{-1}$ W for the 365 nm LED and $(9.335 \pm 0.044) \times 10^{-3}$ W for the 280 nm LED. These values correspond to transmitted fractions of 0.577 ± 0.006% and 0.735 ± 0.012% for the 365 and 280 nm LEDs, respectively.

The beam-profile measurements indicate that part of the incident UV light is intercepted by the trap entrance/lens assembly. No obvious progressive loss of ion signal or the appearance of contamination peaks attributable to charging of these elements was noted over the irradiation times used in this study, although this was not assessed quantitatively here.

Table S1. Optical power measured outside the instrument at approximately 13 cm from the final focusing lens and after transmission through the ion-trap region. Values are background corrected.

LED wavelength	Power outside instrument at ~13 cm	Power after ion-trap region	Transmitted fraction
**365 nm**	$(1.3219 \pm 0.0038) \times 10^{-1}$ W	$(7.62 \pm 0.08) \times 10^{-4}$ W	$0.577 \pm 0.006\%$
**280 nm**	$(9.335 \pm 0.044) \times 10^{-3}$ W	$(6.86 \pm 0.11) \times 10^{-5}$ W	$0.735 \pm 0.012\%$

Irradiation-Time-Resolved FMN Experiments

To probe the irradiation-time dependence of LED-UVPD, FMN was examined using both the 365 and 280 nm LEDs over irradiation times extending from sub-second exposure to 60 s (Figures S2 and S3).

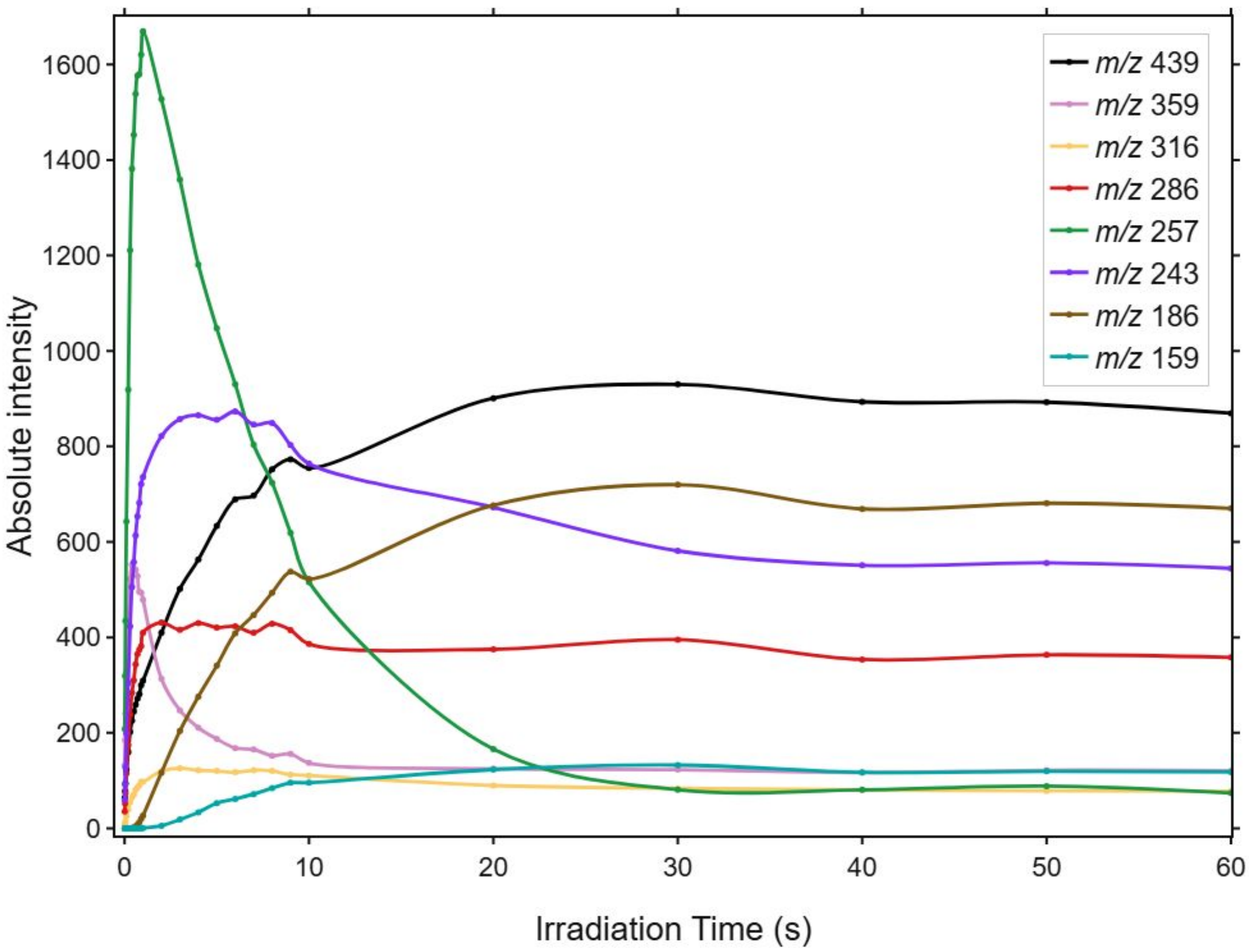

Figure S2. Fragment-ion intensity versus irradiation time for selected FMN photoproduct ions using the 365 nm LED.

For the 365 nm diode (Figure S2), *m/z* 257 and 359 are most intense at shorter irradiation times, while *m/z* 243 increases over the first few seconds and then decreases at longer irradiation times. In contrast, *m/z* 439 and 186 increase more gradually and reach approximately constant intensities after 20-30 s, while *m/z* 159 becomes more prominent at longer irradiation times. These changes in relative fragment intensity may suggest that secondary photofragmentation occurs during extended 365 nm

irradiation. For the 280 nm diode (Figure S3), the major photofragment ions generally increase in intensity with irradiation time over the 60 s period, with less evidence of secondary photofragmentation. The different time-dependent behavior observed for the two LEDs is likely to reflect both the lower optical power delivered at 280 nm (Table S1) and the different electronic transitions accessed at the two excitation wavelengths.

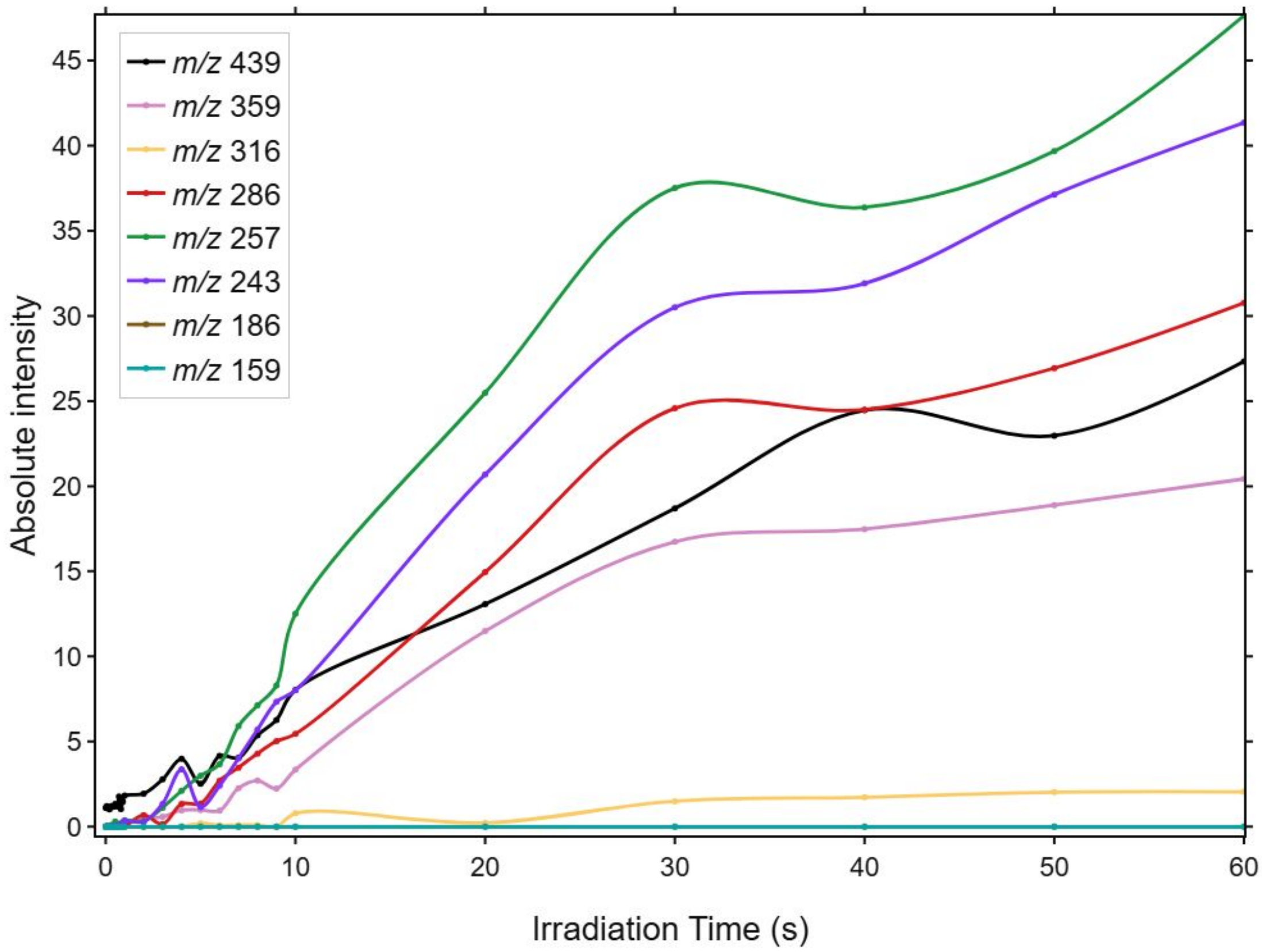

Figure S3. Fragment-ion intensity versus irradiation time for selected FMN photoproduct ions using the 280 nm LED.

Data processing

Mass spectra were exported as text files containing *m/z* and ion-intensity values. Figures were prepared using a custom mass-spectrometry data-processing and plotting application.

Table S2: Fragment ions obtained from CID (MS/MS) and LED-UVPD (365 and 280 nm) for protonated FMN (*m/z* 457.1). Assignments follow Ref [2].

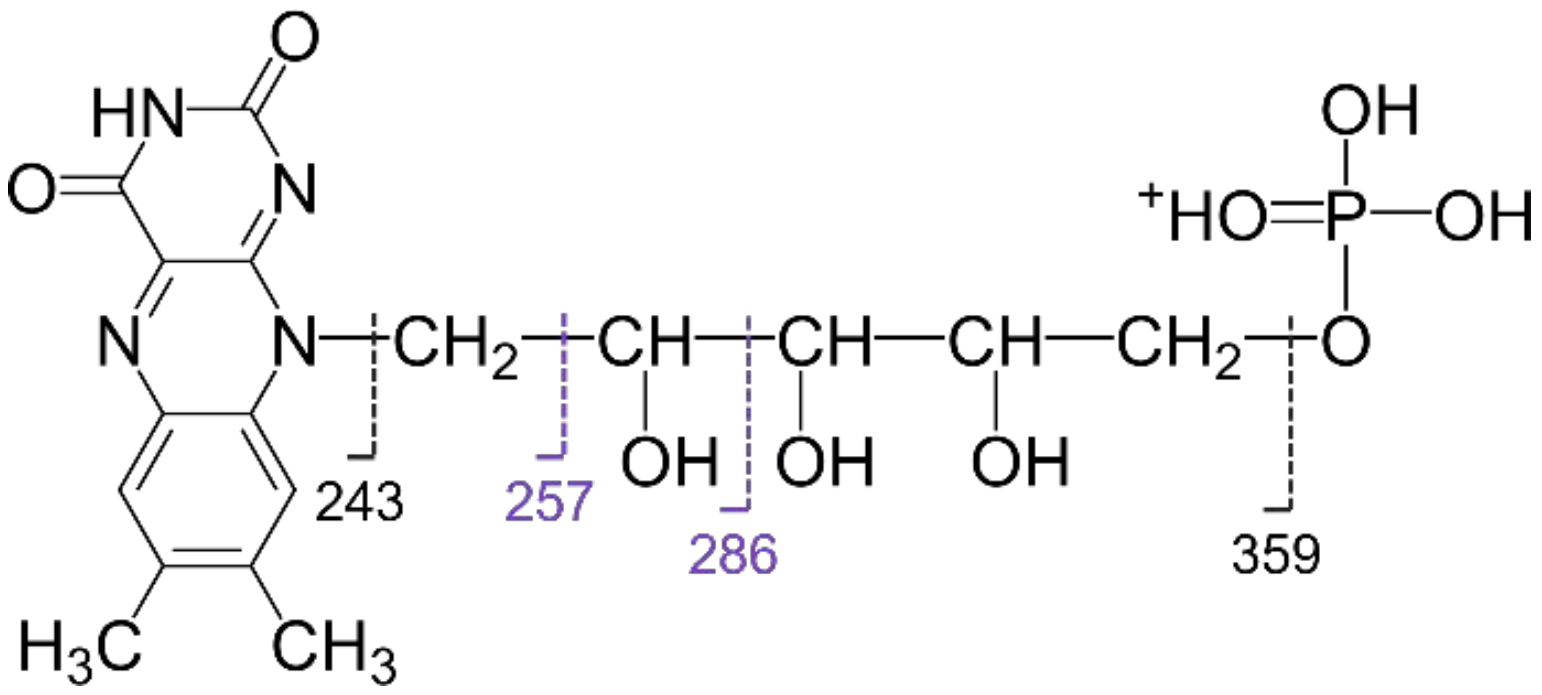

CID (*m/z*)	LED-UVPD 365 nm (*m/z*)	LED-UVPD 280 nm (*m/z*)	Assignment
439.1	439.1	439.2	$[M+H-H_2O]^+$
—	421.1	—	$[M+H-2H_2O]^+$
414.1	—	—	$[M+H-HNCO]^+$ (loss of isocyanic acid)
359.2	359.2	359.2	loss of H_3PO_4 (98 Da)
—	341.1 / 342.1	341.1	loss of H_3PO_4 + H_2O
—	323.1	—	—
—	316.1	316.2	—
—	286.1	286.1	formylmethylflavin
—	257.1	257.1	lumiflavin
243.1	243.1	243.1	lumichrome (loss of ribityl-phosphate side chain)
—	214.1	—	—

—	186.1	—	—
—	172.1	—	—
—	159.1	—	—

Table S3: Comparison of fragment ions observed for protonated FMN by Brodbelt and co-workers (Ref [3]) and this work using CID (MS/MS) and LED-UVPD (365 and 280 nm).

m/z	Brodbelt HCD	Brodbelt LED-UVPD	Our CID	Our LED 280 nm	Our LED 365 nm
**439.1**	✓	✓	✓	✓	✓
**359.1**	✓	✓	✓	✓	✓
**341.1**	—	✓	—	✓	✓
**316.1**	—	✓	—	✓	✓
**286.1**	—	✓	—	✓	✓
**257.1**	—	✓	—	✓	✓
**243.1**	✓	✓	✓	✓	✓
**214.1**	—	✓ extended time	—	—	✓
**186.1**	—	✓ extended time	—	—	✓
**172.1**	—	✓ extended time	—	—	✓
**159.0**	—	—	—	—	✓

Table S4: Comparison of fragment ions reported by Molina et al. (Ref [4]) with those observed in this work using CID (MS/MS) and LED-UVPD (280 nm) of protonated tryptophan (*m/z* 205.1). Fragment assignments follow Ref [4].

**CID (*m/z*)**	**LED 280 nm (*m/z*)**	**Pulsed laser UVPD[b]**	**Assignment[a]** 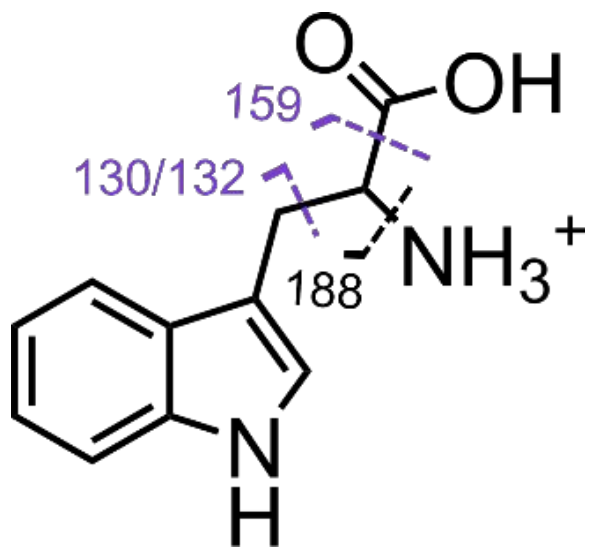
188.0	188.1	✓	$[Trp+H-NH_3]^+$
—	170.0	—	UVPD product; ? $[Trp+H-H_2O\text{-}NH_3]^+$
159.1	159.1	✓	Trp immonium ion / $[M+H-CO-H_2O]^+$
—	146.0	✓	$[Trp+H-NH_3-CH_2CO]^+$
—	144.1	—	—
132.1	132.0	✓	Cα–Cβ cleavage product
—	130.1	✓	Cα–Cβ cleavage product; indolylmethyl/3-methyleneindolenium-type ion
118.1	118.1	—	secondary fragmentation product; unassigned

[a] Purple lines depict UVPD fragmentation, black lines CID.

[b] REF [4].

Minor differences are evident between the 280 nm LED-UVPD fragments and those observed in previous pulsed laser photodissociation. There may be a number of reasons for this, including trapping and detection times, as well as differences in excitation wavelength.

Table S5: Comparison of fragment ions reported by Herburger et al. (Ref [5]) with those observed in this work using CID (MS/MS) and LED-UVPD (280 nm) of protonated Leu-Enk, $[YGGFL+H]^+$ (*m/z* 556.3). Fragment assignments follow Refs [5–7].

**CID (*m/z*)**	**LED 280 nm (*m/z*)**	**Pulsed laser UVPD (225-300 nm)[a]**	**Assignment** 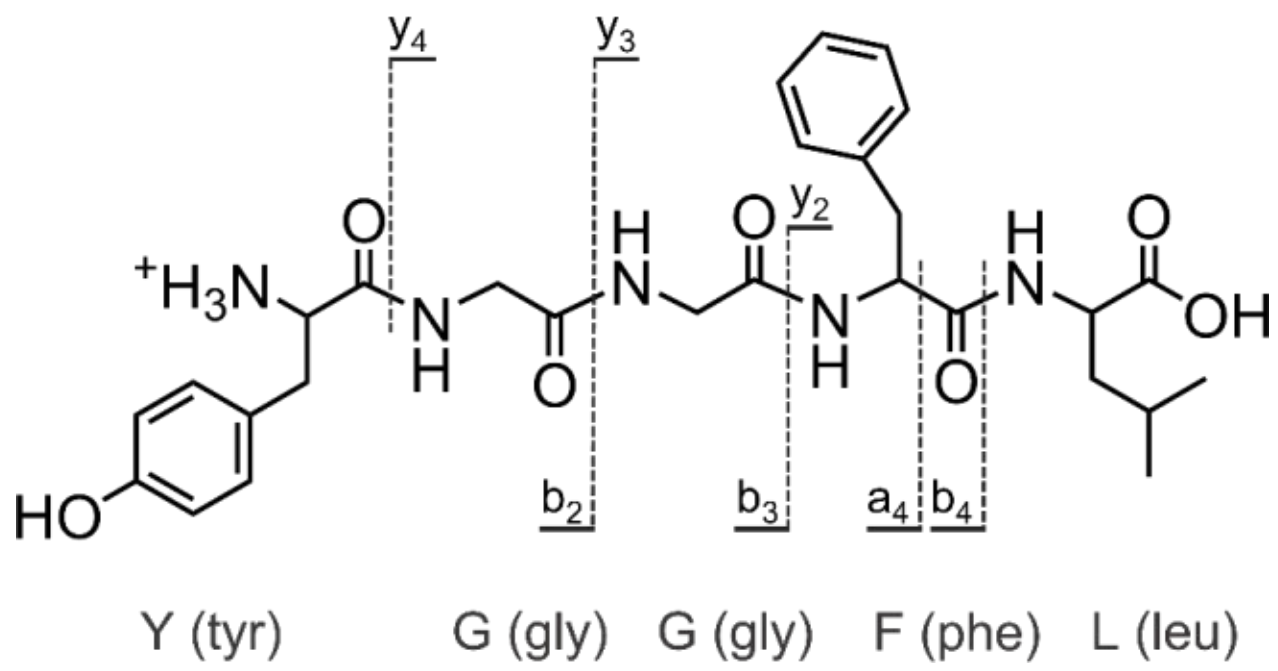
539.1	539.2	—	$[M+H-NH_3]^+$
538.2	538.2	✓	$[M+H-H_2O]^+$
—	521.2	—	$[M+H-NH_3-H_2O]^+$
510.2	510.6	✓	$[M+H-HCOOH]^+$
493.1	—	—	$[M+H-NH_3-HCOOH]^+$
465.2	465.1	✓	$[M+H-FSC]^+$ / $[M+H-91]^+$
—	449.1	✓	$[M+H-YSC]^+$ / $[M+H-107]^+$
425.0	425.2	✓	b_4
—	408.0	—	b_4-NH_3
397.1	—	✓	a_4

393.2	—	—	y_4
380.1	—	—	a_4-NH_3
375.1	375.1	✓	y_4-H_2O
336.1	336.1	✓	y_3
323.1	—	—	a_4-NH_3-G
318.1	318.1	✓	b_4-YSC (Y tyrosine, SC side chain)
—	290.1	✓	a_4-YSC (Y tyrosine, SC side chain)
279.2	—	—	y_2
278.1	—	—	b_3
262.0	—	—	y_2b_4
221.0	—	—	b_2
205.0	—	—	y_3b_4
177.1	—	—	y_3a_4

[a] Ref [5].

Minor differences are evident between the 280 nm LED-UVPD fragments and those observed in previous pulsed laser photodissociation. There may be a number of reasons for this, including trapping and detection times in the different instruments, and also differing detection efficiency for lower-intensity fragments.

References

[1] Dang, A.; Korn, J. A.; Gladden, J.; Mozzone, B.; Tureček, F. UV–Vis Photodissociation Action Spectroscopy on Thermo LTQ-XL ETD and Bruker amaZon Ion Trap Mass Spectrometers: a Practical Guide. *J. Am. Soc. Mass Spectrom.* 2019, 30 (9), 1558–1564. https://doi.org/10.1007/s13361-019-02229-z

[2] Bellina, B.; Brown, J. M.; Ujma, J.; Murray, P.; Giles, K.; Morris, M.; Compagnon, I.; Barran, P. E. UV Photodissociation of Trapped Ions Following Ion Mobility Separation in a Q-ToF Mass Spectrometer. *Analyst* 2014, 139 (24), 6348–6351. https://doi.org/10.1039/c4an01656d

[3] Holden, D. D.; Makarov, A.; Schwartz, J. C.; Sanders, J. D.; Zhuk, E.; Brodbelt, J. S. Ultraviolet Photodissociation Induced by Light-Emitting Diodes in a Planar Ion Trap. *Angew. Chem. Int. Ed.* 2016, 55 (40), 12417–12421. https://doi.org/10.1002/anie.201605850

[4] Molina, F.; Dezalay, J.; Tabata, J.-I.; Soorkia, S.; Broquier, M.; Hirata, K.; Ishiuchi, S.-I.; Fujii, M.; Grégoire, G. Conformer-Selective Photodynamics of $TrpH^+{-}H_2O$. *ChemPhysChem* 2023, 24 (2), e202200561. https://doi.org/10.1002/cphc.202200561

[5] Herburger, A.; van der Linde, C.; Beyer, M. K. Photodissociation Spectroscopy of Protonated Leucine Enkephalin. *Phys. Chem. Chem. Phys.* 2017, 19 (17), 10786–10795. https://doi.org/10.1039/c6cp08436b

[6] Dörner, S.; Schwob, L.; Schubert, K.; Girod, M.; MacAleese, L.; Pieterse, C. L.; Schlathölter, T.; Techert, S.; Bari, S. The Influence of the Methionine Residue on the Dissociation Mechanisms of Photoionized Methionine-Enkephalin Probed by VUV Action Spectroscopy. *Eur. Phys. J. D* 2021, 75, 142. https://doi.org/10.1140/epjd/s10053-021-00147-y

[7] Ranković, M. Lj.; Canon, F.; Nahon, L.; Giuliani, A.; Milosavljević, A. R. VUV Action Spectroscopy of Protonated Leucine-Enkephalin Peptide in the 6–14 eV Range. *J. Chem. Phys.* 2015, 143 (24), 244311. https://doi.org/10.1063/1.4939080
